# Follicular and hormonal changes after estrous synchronization in bottlenose dolphins

**DOI:** 10.1530/RAF-22-0039

**Published:** 2022-09-29

**Authors:** Gisele Montano, Pat Clough, Todd Schmitt, Michelle Davis, Karen Steinman, Justine O’Brien, Todd Robeck

**Affiliations:** 1 SeaWorld and Busch Gardens Species Preservation Laboratory, SeaWorld Parks and Entertainment Inc., Orlando, Florida, USA; 2 Dolphin Research Center, Grassy Key, Florida, USA; 3 SeaWorld Parks and Entertainment Inc., SeaWorld California, San Diego, California, USA; 4 SeaWorld Parks and Entertainment Inc., SeaWorld Florida, Orlando, Florida, USA

**Keywords:** reproduction, cetaceans, endocrinology, bottlenose dolphin, folliculogenesis

## Abstract

To gain more knowledge about the influence of hormone regulation on follicle development, ovarian ultrasounds were performed, and urinary hormone profiles were determined in ovulating and non-ovulating female bottlenose dolphins (*n*  = 15) following estrus synchronization with altrenogest. Ovarian ultrasounds were conducted daily, post-synchronization to describe follicular recruitment in relation to the endocrine profile. Follicle sizes were grouped into very small (VSM), small (SM), medium (MD) and large (LG). In ovulating females, two follicular waves were identified, and follicular deviation toward establishing a dominant follicle only occurred during the second wave. For non-ovulating females, only the first wave was observed. For all urinary hormones, the non-ovulating group presented significantly lower concentrations of follicle-stimulating hormone (uFSH), luteinizing hormone (uLH), estrone conjugates (uE1-C) and estriol (uE3) but similar progestagen and cortisol concentrations compared to the ovulating group. Concentrations of uE1-C and uE3 and the numbers of MD and LG follicles significantly (*P* < 0.05) increased, while uFSH concentrations significantly (*P* < 0.05) decreased as ovulation approached. Urinary LH significantly increased concurrently with increasing numbers of LG follicles and decreasing numbers of SM follicles. The characterization of follicular development and its relationship with hormone assessment complements our understanding of follicular recruitment post-synchronization in bottlenose dolphins and provides new information concerning differences between ovulating and non-ovulating females in response to an estrous synchronization protocol.

**Lay summary:**

Many species of fresh and saltwater dolphins and porpoises are facing increasing pressure for food and habitats due to human activities. One of the primary aspects that can be affected by such activities is reproduction. The bottlenose dolphin has been successfully bred in zoos and aquariums for at least 70 years, and they can be trained for several voluntary behaviors that enable medical examinations. Therefore, they represent a unique resource for research and understanding of normal dolphin reproduction. In this research, voluntary ultrasound exams and urine samples from 15 female dolphins were used to describe changes in their ovaries. The resulting descriptions and comparisons provide insight into the unique ovarian physiology of bottlenose dolphins and into the differences between animals after treatment. This information adds to the body of knowledge which one day may be used for developing advanced reproductive techniques to help preserve endangered species of dolphins.

## Introduction

The use of serial ultrasonography combined with simultaneous endocrine profiling has resulted in a better understanding of follicular wave dynamics in domestic animals (Ginther *et al.* 1989, Bergfelt *et al.* 1991, Ginther & Kot 1994, Rubianes & Menchaca 2003, Jaiswal *et al.* 2004). This knowledge has facilitated the improvement of methods used for estrous cycle manipulation in natural or assisted breeding programs, and it can be integrated into species’ reproductive health assessment program in order to help diagnose abnormalities in reproductive function, determination of pregnancy and detection of senescence.

Cetaceans under human care can be trained for a variety of behaviors that help facilitate veterinary care, and among these behaviors is transabdominal ultrasound. Although this tool has been previously used to describe ovarian activity in bottlenose dolphins, *Tursiops truncatus* (Brook 1997, Robeck *et al.* 1998, 2005, Muraco *et al.* 2009), the relationships between follicular wave dynamics and profiles of commonly monitored reproductive hormones have not been adequately described for this species.

Bottlenose dolphin females are classified as facultatively polyestrous with seasonal trends and a cycle length of 33 days (range: 31–36; (Robeck *et al.* 2018). However, the irregularity of their cyclicity makes the reliability of monitoring natural estrous cycles for timed natural or artificial breeding difficult, and as a result, a synchronization methodology using progestagen suppression of follicular activity has been developed. The use of oral altrenogest or medroxyprogesterone acetate is common in dolphin facilities for temporary suppression of ovarian activity (Robeck *et al.* 2005, 2013, Katsumata 2010). Altrenogest is a synthetic progesterone analog and has been widely used in horse reproduction for more than 45 years (Squires *et al.* 1979, Allen *et al.* 1980). In addition, estrus synchronization or suppression with the use of oral altrenogest has been achieved in domestic pigs (Kraeling *et al.* 1981), domestic cats (Stewart *et al.* 2010) and other exotic (Micheletti *et al.* 2015, Crosier *et al.* 2017, Herrick 2019) and cetacean species (Robeck *et al.* 2004, 2005, 2009, 2010).

For bottlenose dolphins, estrous synchronization with altrenogest results in ovulation approximately 20 days post-withdrawal (Robeck *et al.* 2005). Although this is the primary synchronization protocol currently used for bottlenose dolphins, its effectiveness is a major limitation in that only approximately 50% of animals placed on the hormone will respond to it by ovulating (Robeck *et al.* 2013).

Quantitative measurement of luteinizing hormone (LH) in bottlenose dolphins was first reported using radioimmunoassay of sera (Schneyer *et al.* 1985, Yoshioka *et al.* 1986). This method was validated for its use in urine to help characterize the estrous cycle of the killer whale (Robeck *et al.* 1993, 2005), and different LH-specific RIAs were also used to determine serum concentrations of the hormone for the Yangtze finless porpoise (*Neophocaena phocaenoides asiaeorientalis*).

Measurement of delphinid urinary estrogen conjugates by EIA was first validated in killer whales and later applied to bottlenose dolphins using an antibody with high cross-reactivity to estrone conjugates (uE1-C), that is, estrone-3-glucuronide and estrone-3-sulfate (E1-S) (Robeck *et al.* 2004, 2005). As with many other mammalian species, estradiol (E2) is assumed to be the major bioactive estrogen in delphinids, with serum circulating concentrations increasing ~ten-fold during estrus from baseline to preovulatory concentrations in the bottlenose dolphin (Yoshioka *et al.* 1986). Estriol (E3) is considered a less bioactive form of estrogen with increased concentrations typically associated with placental aromatase activity (Ryan 1982).

Folliclestimulating hormone (FSH) is a relatively conserved pituitary peptide associated with follicular recruitment in mammalian species (reviewed by Suresh *et al.* 2011). In cetaceans, serum FSH was first analyzed using RIA in random samples of bottlenose dolphins (Schneyer *et al.* 1985). Studies on killer whales demonstrated a bimodal pattern of FSH in the follicular phase in killer whale urine samples (Walker *et al.* 1988, Robeck *et al.* 1993). In free-ranging animals, RIA has been used to analyze circulating FSH concentrations in Yangtze finless porpoise (Hao *et al.* 2007).

Plasma progesterone concentrations were first validated and measured in bottlenose dolphins following ovulation induction in 1975 using a competitive protein binding assay (Richkind & Ridgway 1975). Using RIA (Sawyer-Steffan *et al.* 1983), circulating progesterone was measured in bottlenose dolphin serum for 2 years to establish the baseline and reference for this species. RIA was first used to measure urinary progestagens in the false killer whale (*Pseudorca crassidens*) and the killer whale (Robeck *et al.* 1994, 2004). Enzyme immunoassays have also been validated for quantifying urinary progestagens in bottlenose dolphins, Pacific White-sided dolphin (*Lagenorhynchus obliquidens*) and beluga (*Delphinapterus leucas*) (Robeck *et al.* 2005, 2009, Steinman *et al.* 2012).

Cortisol concentrations have been recently described during normal pregnancies in the bottlenose dolphin and killer whale (Steinman *et al.* 2016, Robeck *et al.* 2017), and changes in concentrations have become an important indicator of relative stress and well-being in captive and wild marine mammal species (Fair *et al.* 2014, Serres *et al.* 2020, Nabi *et al.* 2021, Steinman & Robeck 2021). Given the potential relationship of cortisol measurements to stress and reproductive suppression (Moberg 1985, Dobson & Smith 1995, Breen & Karsch 2006), no direct hormone analysis has been studied within a controlled group of cetaceans that have or have not responded to estrus synchronization protocols. In killer whales, evidence suggests that estrus causes a transient increase in cortisol in females that have conceived naturally (Robeck *et al.* 2017).

Despite all aforementioned data on follicular growth, estrogens, LH and FSH in cetaceans, no research has been published that describes the temporal association between the profiles of these hormones and follicular wave development, including follicular recruitment and growth until the establishment of follicular dominance and ovulation. The overall goal of this research was to describe the ovarian and endocrine response to an exogenous progestagen synchronization protocol in an effort to understand the differences between bottlenose dolphin females that do or do not respond to treatment by ovulating. The specific objectives were to (i) describe follicular recruitment, development and primary follicular deviation in female bottlenose dolphins that ovulate post-altrenogest treatment; (ii) describe and compare excretory profiles of urinary progestagens, uE1-C, E3, FSH, LH and cortisol in females that do and do not respond by ovulating post-altrenogest withdrawal and (iii) provide biologic validation of hormonal activity by correlating concentrations with follicular dynamics during the estrous cycle.

## Materials and methods

### Ethics of experimentation

All samples were collected as part of routine husbandry procedures for bottlenose dolphins. All procedures described within were reviewed and approved by each institution’s Animal Care and Use Committee and were performed in accordance with the Animal Welfare Act for the care of marine mammals.

### Animals

Bottlenose dolphin females (*n*  = 15) with a mean age of 17 years (median: 15.5, range: 7–30 years) and weighing at least 180 kg were located at Discovery Cove, Orlando (*n*  = 9), Dolphin Research Center (*n*  = 2) and SeaWorld San Diego (*n*  = 4). Samples were taken during the spring and fall of 2011, fall of 2012, fall of 2013 and winter of 2014. Animals from SeaWorld were housed in pools containing ≥850 m^3^ or naturally processed salt water (ambient temperatures: 14–28**°**C). Animals from Dolphin Research Center were housed in various natural saltwater interconnected enclosures ranging in size from 300 to 600 m^3^ (ambient temperature: 10–30°C). Dolphins were fed approximately 4–5% of their body weight per day, a diet of frozen-thawed whole fish (herring, *Clupea harengus*, capelin, *Mallotus villosus* and Columbia River smelt, *Thaleichthys pacificus*).

### Synchronization of ovulation and artificial insemination

Females did not have access to males for at least 1 month prior to study. To control the day of ovulation for AI scheduling purposes, estrus was synchronized using the synthetic progestin, altrenogest (Regu-Mate®, Intervet Inc., Millsboro, Delaware 19966, USA). All female dolphins were administered altrenogest (0.044 mg/kg p.o.) once daily for 20 days (O’Brien & Robeck 2006, Robeck *et al.* 2013). The drug was given orally by injection directly into the coelomic cavity of a fish just prior to feeding. Serum progesterone was determined at the start of altrenogest to ensure that none of the females had ovulated.

Artificial inseminations (*n*  = 11) were performed on all females that developed a preovulatory follicle as previously described (Robeck *et al.* 2013). Briefly, animals were initially administered diazepam (0.1–0.2 mg/kg p.o.; Abbott Lab) 1 hour prior to the procedure. Animals were then placed in lateral recumbency on 10.2 cm thick closed cell foam pads. Vital signs were monitored, and the animals were kept wet throughout the procedure. A flexible endoscope (9–11 mm in diameter and 120–250 cm long, specifics depend on availability in each facility) was then introduced into the cranial vagina and insufflated with air to improve cervical visualization. After passing the cervix, semen was deposited into the uterine body and/or uterine horn(s) through a catheter (5 or 7 fr, 190–400 cm long, Cook Vet Supplies, QLD, Australia) placed in the working channel of the endoscope.

### Ovarian ultrasonography

Transabdominal ultrasonography was used to detail follicular activity following altrenogest synchronized cycles and to confirm pregnancy as previously described for bottlenose dolphins (Brook 2001, Robeck *et al.* 2005). Ultrasonographic examinations (*n*  = 230) were performed using either an Aloka 900 machine (Corimetrics Medical, Charlotte, NC), GE Logibook™ Book (GE LogiqGE Medical Systems, Milwaukee, WI) or a SonoSite 180 Plus (SonoSite, Inc, Bothell, WA) all with a 3.5–4.0 MHz transducer (wide footprint convex linear probe). For examination, the animals were trained to station in lateral recumbency adjacent to the edge of the pool. The ovaries were located transabdominally using a previously described technique (Brook 2001). Animals were examined once on the last day of altrenogest (day 0), every other day until day 10 post-altrenogest and then daily until ovulation. For the exam, each ovary was scanned several times using similar methods during each exam and the images were stored in 3 s cineloops for retrospective analysis on the machine or using OsiriX MD software (Pixmeo Labs, Geneva, Switzerland).

**Figure 1:**
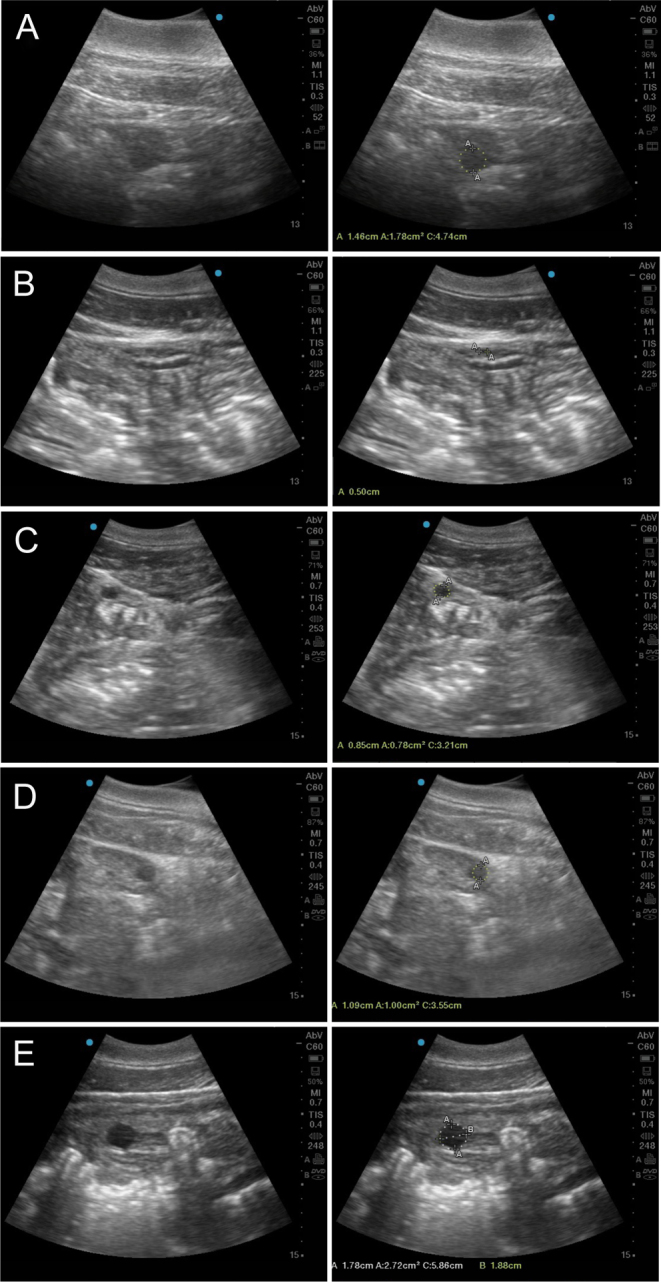
(A) Follicle visualization in an animal (not used in the study) with thick blubber layer to demonstrate how it affects the quality of the image. (B) Very small follicles (3–6 mm). (C) Small follicles (6.1–10 mm). (D) Medium size follicles (10.1–16 mm). (E) Large size follicle (>16 mm).

Follicle diameter (maximum diameter) of each follicle greater than 3 mm was determined, retrospectively, by examining the cineloops one frame at a time. Individual follicle sizes and approximate locations from each daily exam were represented on an ovarian map that facilitated the tracking of individual follicles from one exam to the next. A follicular wave was defined as the synchronous emergence of follicles 3 mm in diameter that, as the wave progressed, at least one of the cohort of follicles continued to grow, while the others regressed at variable intervals. The largest and second largest follicles that emerged (determined retrospectively as any follicle 2 mm or greater in diameter in the remaining group that continued to separate itself in size from the others for at least 4 days in a row) from the cohort of growing follicles were defined as F1 and F2 (Manjunatha *et al.* 2012). For analysis, the day relative to the day prior to ovulation (DPO) when the F1 separated itself from the remaining cohort was termed the day of deviance (DOD). The DOD was considered the point at which the preovulatory follicle established dominance over the remaining cohort (Burns *et al.* 2005). Follicles observed daily included growing, non-growing and atretic follicles and were retrospectively grouped by ovulatory or non-ovulatory ovarian side, actual ovarian side (left or right) and size as follows: very small (VSM: 3–6 mm); small (SM: >6–10 mm); medium (MD: >10–16 mm); and large (LG: >16 mm; Fig. 1).

For a retrospective comparison of ovulatory and non-ovulatory cycles, the estimated DOD for ovulatory cycles was overlaid onto the mean day post-altrenogest at which ovulation occurred. For example, ovulation occurred at a mean of 23 days post-altrenogest with DOD approximately 10 days prior, and therefore, follicular and hormone data from all non-ovulating cycles were collected until 23 days post-altrenogest, with 13 days post-altrenogest becoming the pseudo day of deviance and 23 days post-altrenogest represented the pseudo day of ovulation. For ease of discussion and comparison between this non-ovulating and the ovulation group, the non-ovulating female groups were then aligned, analyzed and discussed based on these estimated DOD and days prior to ovulation, despite the fact that ovulation did not occur.

### Hormone analysis

Urine samples were collected from unrestrained animals as previously described (Lenzi 2000, Robeck *et al.* 2009). Daily urine collections were performed from day 1 to 9 after altrenogest withdrawal, twice a day urine collection (8–12 h apart) started on the 10th day until a dominant follicle was identified by ultrasonography, after which, urine collection was increased to three times a day (6–8 h apart) until ovulation. Each sample was split in two, 2 mL aliquots, one was stored at 4°C and analyzed in batches every 2–3 days for EC and creatinine. The other aliquot was stored at −80°C and analyzed at further date for cortisol, E3, LH, FSH, Pg and re-analysis of EC. During the periovulatory period, a third aliquot of urine samples was used to identify the LH curve using a semi-quantitative LH test kit (Witness Synbiotics Corp., Kansas City, MO, USA) as previously described (Muraco *et al.* 2009, Robeck *et al.* 2009).

#### Creatinine assay

Urine samples were analyzed for creatinine (Cr) to account for varying concentrations of urine as previously described (Taussky & Kurzmann 1954). Concentrations of urinary hormones and metabolites were indexed to Cr and expressed as mass (ng) of hormone per mg Cr excreted.

#### Estrone conjugates

Urinary estrone conjugates (uE1-C) were measured by a single antibody, direct enzyme immunoassay (EIA) as previously described and validated for use with bottlenose dolphin urine (Robeck *et al.* 2005, 2013). Briefly, neat urine samples (0.050–0.0025 mL) and standards (estrone-B-glucuronide, range: 200–0.79 pg/well, Sigma–Aldrich) were added to a microtiter plate coated with antisera (1:20,000, R522-2, C. Munro). An enzyme conjugate label (horse-radish peroxidase (HRP), 1:250,000, C. Munro) was added to all wells and plates were incubated for 2 h at room temperature and then washed before the addition of tetramethylbenzidine (TMB) in phosphate citrate buffer (Sigma–Aldrich) and a final incubation on a plate shaker (400 rpm, MidSci, Valley Park, MO). A stopping solution was then added (0.6 M H_2_SO_4_) and the optical density was measured at 450 nm with a 655 nm reference filter (Model 680, BioRad). Assay sensitivity was 3.9 pg/well and intra-assay variation was <10% and inter-assay variation was 10.1% and 11.9% (*n*  = 71) at 30% and 70% binding, respectively.

#### Urinary estriol

The urinary estriol (uE3) concentrations were measured using a single antibody, direct EIA as previously described (Hedriana *et al.* 2001). This EIA was previously validated for bottlenose dolphin serum (Steinman *et al.* 2016). Neat urine (0.05–0.025 mL) and standard (15.6 to 4000 pg/well) were added to a 96-well microtiter plate coated with a polyclonal antibody (1:10,000, R4835, C. Munro) along with an E3-HRP (1:50,000). Following a 2 h incubation at room temperature, 0.1 mL substrate solution (azino-bis-3ethyl benzthiazoline-6-sulfonic acid (ABTS) in citrate buffer, Sigma–Aldrich) was added and the plate was incubated for another 20–30 min on a plate shaker. The plate was read at 405 nm with a 540 nm reference filter. Reported cross-reactivity for antibody R4835 was specific for E3 with other steroids and metabolites structurally similar to E3 showing <0.1% cross-reaction (Hedriana *et al.* 2001). However, Steinman *et al.* (2016) demonstrated that estradiol had ~67% cross-reactivity with this antibody. Assay sensitivity was 15.6 pg/well and intra-assay variation was <10%. Parallel displacement of a pool of urine compared to the standard curve was demonstrated (r = 0.999, *P* < 0.05) and an accuracy test plotting the concentrations of a pool of urine spiked with known concentrations of standard against non-spiked pool yielded a recovery of 89.4 ± 11.3% (linear regression, y = 0.89x – 14.66, r^2^ = 0.999). Samples were run in duplicate and if the CV between the replicates was >10%, the sample was repeated. Inter-assay coefficients of variation for the two quality controls bounded at 30 and 70% were 6.7 and 8.7%, respectively (*n*  = 28).

#### Urinary luteinizing hormone

A double antibody LH EIA has been previously described and validated for bottlenose dolphin urine (Robeck *et al.* 2005). Briefly, microtiter plates pre-treated with goat anti-mouse IgG (X12-1EA, Arbor Assays, Ann Arbor, MI) were blocked with 0.02 M Trizma buffer (Sigma–Aldrich) and incubated at room temperature overnight or up to 3 weeks. Plates were emptied, washed (0.02% Tween solution, Sigma–Aldrich) and neat urine (0.05–0.001 mL) and standards (bovine LH, NIH-LH-B10, AFP-5551B, National Institute of Health, Bethesda, MD, USA) were added to wells along with 0.1 mL of antibody (monoclonal anti-bovine LH, LH 518-B7, 1:600,000, J. Roser, UC Davis, CA, USA) and incubated at room temperature overnight. Biotinylated LH (1:1,500,000) was then added, and after another incubation (4 h, room temperature), the plates were washed and streptavidin peroxidase solution (1 µL in 24 mL assay buffer, Roche Diagnostics) was added. Following 40 min incubation, TMB substrate was added prior to a final incubation (45–60 min). A stopping solution was then added (0.6 M H_2_SO_4_) and the optical density was measured at 450 nm with a 655 nm reference filter. Intra-assay variation was <10% and inter-assay variation was 13.3 and 12% at 30 and 60% binding (*n*  = 35), respectively.

#### Urinary follicle-stimulating hormone

Analysis of urine samples for FSH was performed at the Endocrine Research Laboratory at the Smithsonian Conservation Biology Institute using an in-house ^125^I RIA as described previously (Brown *et al.* 2004). Briefly, 0.1 mL neat urine and ovine FSH standards (250–0.495 ng/mL oFSH NIADDK-oFSH-17, National Institute of Health) were added to the glass tubes. A rabbit anti-ovine FSH antibody (1:25,000) was then added to all tubes and the assay mixture was incubated overnight at room temperature. The next day, 0.1 mL of ^125^I labeled oFSH (~20,000 cpm/tube) was added to all tubes and incubated overnight. Finally, 1 mL of a second antibody, sheep anti-rabbit gamma globulin (1:300 in PBS containing 5% polyethylene glycol), was added to all tubes and incubated for 1 h. Tubes were then centrifuged (3000 ***g*** for 25 min) and the supernatant was decanted. Remaining pellets in the tubes were counted in a gamma counter (Iso Data 20/20 Series, GMI Inc., Ramsey, MN). Assay sensitivity was 0.5 ng/mL (Brown *et al.* 2004) and a serially diluted pool of urine demonstrated parallelism to the standard curve (r = 0.998). Intra-assay variation was <10% and inter-assay variation was 11.6 and 7.5% (*n*  = 5) for two controls binding at 45 and 75%, respectively.

#### Urinary progestagens

Urinary progestagens (uPg) were measured by a single antibody, direct EIA, as previously described and validated for bottlenose dolphin urine (Robeck *et al.* 2005). Briefly, neat urine samples (0.050–0.01 mL) and standards (progesterone, range: 200–0.79 pg/well, Sigma–Aldrich) were added to a microtiter plate coated with a monoclonal antibody (1:10,000, Cl-425, C. Munro). An enzyme conjugate label (HRP, 1:40,000, C. Munro) was added to all wells and plates were incubated for 2 h at room temperature and then washed before the addition of ABTS substrate and a final incubation was done on a plate shaker for 45–60 min. The optical density was measured at 405 nm with a 540 nm reference filter. Intra-assay variation was <10% and inter-assay variation was 10 and 14 % (*n*  = 71) at 20 and 65% binding, respectively.

#### Urinary cortisol

Urinary cortisol (uCort) concentrations were measured using a single antibody, direct EIA, as previously described (Munro & Lasley 1988) and described in detail for use with bottlenose dolphin sera (Steinman *et al.* 2016). Antibody cross-reactivity and sensitivity information are described in detail elsewhere (Steinman *et al.* 2016). Briefly, dilutions of neat urine containing 0.005–0.001 mL of sample and standards (range: 1000–3.9 pg/well, Sigma–Aldrich) were added to a microtiter plate coated with a monoclonal antibody (1:10,600, R4861, C. Munro). An enzyme conjugate label (HRP, 1:33,000, C. Munro) was added to all wells and plates were incubated for 1 h at room temperature and then washed before the addition of ABTS substrate and a final incubation was done on a plate shaker for 15 min. The optical density was measured at 405 nm with a 540 nm reference filter. Parallel displacement of a pool of urine compared to the standard curve was demonstrated (r = 0.998, *P* < 0.05) and an accuracy test plotting the concentrations of a pool of urine spiked with known concentrations of standard against non-spiked pool yielded a recovery of 105.42 ± 8.96% (linear regression, y = 1x + 4.78, r^2^ = 0.999). Inter-assay coefficients of variation (CV) for two quality controls binding at 30 and 70% were 7.3 and 9.5%, respectively (*n*  = 19).

### Statistical analysis

Unless otherwise stated, all statistical analyses were done using STATA (Stata Corp., Version 14). For analyses, all daily observations (follicle data and urinary hormone concentrations) were aligned as either the day post-altrenogest or the day prior to ovulation (DPO). For follicular wave analysis, a maximum likelihood (ML) linear mixed model (LMM) regression analysis (West *et al.* 2014) with animal id as the repeated measures random variable was run separately for the total numbers of follicles within each size group (VSM, SM, MD, LG) for each day post-altrenogest as the dependent variable against the main effects of ovary (ovulating and non-ovulating ovary), rate of increase in follicular numbers within each group day post-altrenogest, the size of the developing preovulatory follicle (POF) and interactions. In addition, a restricted maximum likelihood LMM was also performed with total follicular numbers from both ovaries within each size category DPO as the dependent variable against the independent factorial variables pre- and post-DOD (coded as 0 for pre- and 1 for post-DOD) and ovulation (coded 0 for ovulating females and 1 for non-ovulating females) and their interactions with the random variable animal id. Multiple pairwise ML LMM, with animal id as the random variable, was conducted to determine if follicle numbers within each size category correlated against the other categories (e.g. VSM vs SM follicles) before or after DOD while accounting for the repeated measures characteristics of the data. If the analysis indicated a significant effect, then a relative comparison of goodness of fit between each pair was conducted to determine marginal (mR^2^) and conditional r-squared (cR^2^) using a mixed model analysis (Nakagawa & Schielzeth 2013) with the MuMLN package (lme4) (Bates *et al.* 2014). A similar analysis for pairwise correlation of LMM with mR^2^ and cR^2^ was used to determine if any significant correlations (*P* ≤ 0.05) existed between urinary hormones and follicle size across all time points prior to ovulation (only within ovulating females), and within urinary hormones. Finally, bivariate pairwise correlation (two-tailed) was used to determine if any significant relationship existed between the age of animal and the peak number of follicles within each group associated with each individual animal during each estrus event and the size of the POF, respectively.

Marginal means from each group were compared using pairwise comparisons with a Sidak correction factor. All final mixed models were checked for normality using quantile plots of the standard residuals. If quantile–quantile plots of standardized residuals exhibited non-normal distribution and then data were transformed as recommended by the Shapiro–Wilk test (Ladder command, STATA) until the residuals were normalized.

## Results

Of the 19 (within 15 animals) estrus synchronization attempts in this study, 11 (58%) resulted in ovulation with 4 (36%) becoming pregnant after AI. A total of 230 ovarian ultrasound exams (Fig. 1) were conducted over these 19 cycles (average: 14.77 and 8.5 exams per cycle for ovulating and non-ovulating females, respectively). For one female, due to poor resolution of her ovaries during ultrasound exams, only POF determination was possible. This was believed to be due to her large s.c. fat layer (>2.8 cm) which attenuated the image quality (Robeck 1996).

### Follicular growth characteristics in animals that ovulated

**Figure 2:**
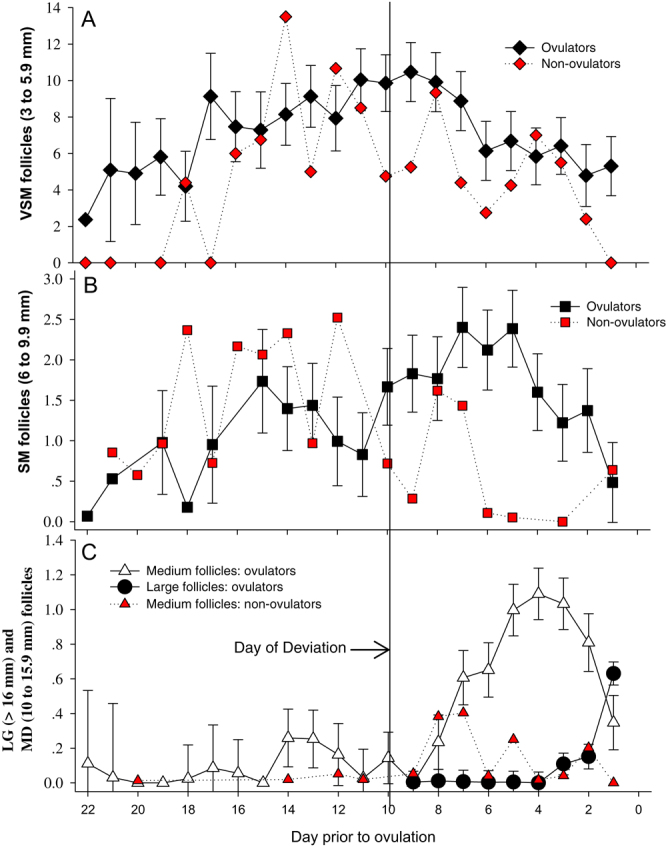
Daily follicular counts from both ovaries within each size group from ovulators (black or white) and non-ovulators (red). (A) Cohort of very small follicles (3–6 mm) with peak at day 17 prior to ovulation. (B) For ovulators, the wave peak for small follicles (6.1–10 mm) occurred on days 15 and 7 prior to ovulation. (C) Medium-size follicles (10.1–16 mm) peaked on day 14, followed by a second wave that commenced on day 8 prior to ovulation and developed into the large follicle (>16 mm).

For animals that ovulated, we observed a relatively large cohort (>5) of very small follicles with two waves of small follicles detected, and the dominant follicle was identified from the second wave prior to ovulation. The two waves of small follicles began on days 18 and 11 prior to ovulation and peaked on days 15 and 7, respectively (Fig. 2B). During the second wave of small follicles, only one follicle was identified as having separated itself from the remaining cohort of follicles. In all cases, this follicle was the dominant follicle (F1) that eventually ovulated. The day of deviance (DOD) was established with a mean of 9.7 days (±0.74 days) prior to ovulation and a mean size of 7.1 ± 0.4 mm in diameter. From the DOD, the F1 follicle grew at a rate of 1.3 ± 0.2 mm/day reaching a mean size of 18.4 ± 0.6 mm at 12–24 h prior to ovulation. A steady mean daily growth rate was observed until the day prior to ovulation, when it doubled to 24 mm/day. When excluding F1 from the total follicular counts, no significant differences were detected between total follicle numbers on contra or ipsilateral (to POF) ovaries in the VSM (*P* = 0.31), SM (*P* = 0.79), MD (*P* = 0.43) or LG (no follicles) groups; therefore, for all further analysis, follicle numbers from both ovaries within each group were combined to represent total follicle numbers (T).

A significant negative effect (β_2_ = −3.14, *P* = 0.008) was detected between the size of the F1 follicle and total VSM numbers (Fig. 1). VSM follicles were influenced by DPO (*P* < 0.001) and had significantly different (*P* = 0.04) total numbers before and after dominance (DOD) was established. In addition, VSM follicle numbers had a linear relationship with DPO (*P* < 0.001) where they increased at a rate of 0.49 follicle/DPO until the day of deviation after which they decreased linearly (*P* < 0.001) at a rate of 0.56 follicle/DPO (Fig. 2A). Overall, SM follicles had no relationship with DPO (*P* = 0.477), but total SM follicle numbers were reduced (*P* = 0.01) before vs after dominance was established (Fig. 2B). MD follicles were associated with DPO (*P* < 0.001), but total follicle numbers were not different (*P* = 0.71) when DPO was included as a factor in the analysis. A positive association (*P* < 0.001) was detected between the numbers of MD follicles and DPO after dominance was established but not before (*P* = 0.39). As expected, when simply comparing follicle numbers before vs after DOD (without controlling for DPO), MD follicles were greater (*P* = 0.0001) after DOD (Fig. 2C). Large follicle numbers increased with DPO (*R^2^* = 0.25, *P* < 0.001, Fig. 2C). Although no significant relationship was detected between the follicular size groups, there appeared to be an initial wave of follicles that occurred prior to the emergence of the F1 follicle or DOD (Fig. 2A, B and C). This wave peaked prior to regression at DPO of 17 for VSM, at DPO of 15 for SM and at DPO of 14 for MD follicles.

After dominance was established, significant correlations existed between total (t) VSM and tSM (*mR^2^* = 0.011, cR^2^ = 0.66, *P* = 0.03) follicles, tVSM and tLG (*mR^2^*= 0.03, cR^2^ = 0.60, *P* = 0.001) follicles, tSM and tLG (*mR^2^* = −0.02, cR^2^ = 0.59, *P* = 0.05) and tMD and tLG (*mR^2^* = −0.15, cR^2^ = 0.42, *P* < 0.0001) follicles. These correlations can be visualized (Fig. 2A, B and C) and represented the second or final follicular wave prior to ovulation. During this final wave, peak VSM numbers occurred on day 9, peak small follicle numbers on day 7 and medium number of follicles on day 4 prior to ovulation. As age increased, so did the peak number of small (*r* = 0.67, *P* = 0.03) and medium follicles (*r* = 0.63, *P* = 0.049), but no significant correlation was detected between age and very small (*r* = 0.31, *P* = 0.38) or the size of the POF (*r* = −0.29, *P* = 0.39). No difference was detected in F1 growth rates between animals that did (*n*  = 4) or did not conceive (*n*  = 7, *R*^2^ = 0.10, *P* = 0.33). However, animals that produced a term calf (*n*  = 3) had a shorter period of time from peak VSM to ovulation (8 ± 0.7 days vs 10.4 ± 0.5 days, *P* = 0.02) and an increased F1 growth rate (1.8 ± 0.2 mm day vs 1.1 ± 0.2 mm/day, *P* = 0.043) compared to ones that did not produce a live calf, a group that included those that failed to conceive (*n*  = 7) and one that was pregnant but experienced an early embryonic loss previously defined as <day 120 post ovulation (O’Brien & Robeck 2012).

### Follicular patterns in ovulating vs non-ovulating female dolphins

Within all follicle size groups, non-ovulatory females had significantly reduced mean numbers of follicles observed post-altrenogest treatment (Table 1). This also held true when follicular growth patterns were partitioned between pre- and post-day of deviance (Table 1). Initially, the pattern of follicular development for animals that did not ovulate appeared similar to ovulators with an initial development of VSM that peaked on day 14 vs day 17 DPO (Fig. 2A). This peak was then followed by SM follicles that peaked at day 12 DPO, with the second peak on 8 DPO. Similarly, MD follicles peaked on day 7 but regressed quickly, without the formation of an obvious F1 follicle by day 6 (Fig. 2C). The similarities in follicular patterns within the VSM and SM follicle cohorts between the non-ovulatory female group and the ovulatory female group were largely attributed to a subset of females (*n*  = 3) within the non-ovulatory group designated as initially responding (to synchronization protocol) non-ovulatory females.

**Table 1:** Developmental comparisons of very small follicles (VSM), small follicles (SM) and medium follicles (MD) from both ovaries between animals that ovulated (*n*  = 11) and ones that did not ovulate (*n*  = 7) before and after day of deviance (DOD).^1^ Data are presented as total follicle numbers (marginal means (95% CI).

	VSM: 3−6 mm	SM: 6−10 mm	MD: 10−16 mm
Post-regumate to ovulation			
Ovulatory (A)	8.8 (6.0−11.5)	0.86 (0.44−1.42)	0.78 (0.70−0.87)
Non-ovulatory (B)	1.2 (0−4.1)	0.18 (0.013−0.53)	0.52 (0.44− 0.62)
Mean comparisons^*^	B < A	B < A	B < A
Ovulators			
Prior to DOD (A)	10.7 (6.7−14.8)	0.74 (0.18−1.68)	0.57 (0.5−0.71)
After DOD (B)	7.3 (5.1−9.5)	0.96 (0.53−1.51)	0.92 (0.79−1.01)
Non-ovulators			
Prior to DOD (C)	0.96 (0−5.3)	0.26 (0.0−1.0)	0.49 (0.39−0.60)
After DOD (D)	1.4 (0−3.9)	0.12 (0.0−0.42)	0.55 (0.43−0.69)
Mean comparisons^*^	C & D < A & B	D < B	A & C & D < B
Initial responders (*n* = 3)			
Prior to DOD (A)	5.17 (1.78−10.32)	1.39 (0.94−1.84)	0.69 (0.62−0.76)
After DOD (B)	5.22 (3.63−7.11)	1.02 (0.65−1.40)	0.77 (0.71−0.84)
Non-responders (*n* = 4)			
Prior to DOD (C)	0.33 (0.15−2.34)	0.32 (0−0.68)	0.71 (0.67−0.76)
After DOD (D)	0.34 (0.04−0.95)	0 (0−0.34)	0.79 (0.71−0.87)
Mean comparisons^*^	D & C < B & A	D < B & A; C < A	

^1^DOD for animals that did not ovulate was determined by overlaying the DOD (day 10 prior to ovulation) and the mean days post-regumate that animals ovulated (day 23 post-regumate). The F1 follicle was removed from the comparison;.^ *^Only means within each group (values prior to the mean comparison) within each column that were significantly different (*P* ≤ 0.05), with Sidak correction, are listed.

The initially responding non-ovulating females (IR) had several characteristics that differentiated themselves from the remaining non-ovulatory, non-responding females (*n*  = 4) including: (1) VSM and SM follicle groups were significantly reduced (*P* < 0.05) for the non-responding, non-ovulatory females compared to the initial responder females before and after the estimated day of deviance (Tables 1 and 2) and (2) initial responding females had mean ovarian VSM and SM follicle counts that were either similar (VSM) or significantly greater than (SM) ovulating females prior to the DOD (Table 1).

**Table 2:** Comparisons of urinary estrone conjugate (E1-C), estriol (uE3), follicle-stimulating hormone (uFSH), luteinizing hormone (uLH), progestagens (uPg) and cortisol (uCort) concentrations (ng/mg creatinine) between animals that ovulated (*n*  = 11) and ones that did not ovulate (*n*  = 8) before and after day of deviance (DOD).^1^ Data are presented as marginal mean hormone concentrations (95% CI).

	E1-C	uE3	uFSH	uLH	uPg	uCort
Ovulators						
Overall^2^ (A)	1.4 (1.1−1.7)	3.9 (2.6−5.7)	10.8 (8.3−14.0)	4.6 (3.4−6.0)	0.45 (0.40−0.53)	16.5 (14.0−19.2)
Prior to DOD (B)	0.9 (0.7−1.0)	3.3 (2.3−4.9)	9.6 (7.3−12.6)	3.1 (1.8−4.7)	0.45 (0.37−0.56)	15.5 (12.7−18.5)
After DOD (C)	2.0 (1.6−2.5)	4.5 (3.0−6.6)	11.8 (9.0−15.4)	6.4 (4.5−8.6)	0.46 (0.41−0.51)	17.4 (14.6−20.3)
Non-ovulators						
Overall (D)	0.8 (0.6−0.9)	1.3 (0.8−2.0)	6.9 (5.2−9.1)	2.3 (1.8−2.8)	0.50 (0.36−0.69)	17.3 (15.3−19.4)
Prior to DOD (E)	0.8 (0.6−0.9)	1.4 (0.9−2.1)	7.0 (5.2−9.5)	3.7 (2.8−4.6)	0.50 (0.31−0.81)	17.9 (15.7−20.3)
After DOD (F)	0.8 (0.6−1.0)	1.2 (0.8−1.9)	6.8 (5.1−9.0)	1.3 (0.7−1.9)	0.50 (0.39−0.64)	16.7 (14.6−19.0)
Mean comparisons^*^	D < A; E, F & B < C	D < A; E & F < B < C	D < A; E & F < B < C	D < A; F< B & E < C		

^1^DOD for animals that did not ovulate was determined by overlaying the DOD (day 10 prior to ovulation) and the mean days post-regumate that animals ovulated (day 23 post-regumate); ^2^Overall: hormone concentration from the day of regumate withdrawal until ovulation; ^*^Only means within each column that were significantly different (*P* ≤ 0.05), with Sidak correction, are listed.

### Urinary endocrine patterns in ovulating vs non-ovulating female dolphins

Because no significant differences (*P* < 0.05) were detected in urinary hormone concentrations among non-ovulating, initial responding females or non-responding female groups, the data were combined for comparison against the ovulatory group. Across all days prior to ovulation (or post-altrenogest for non-ovulators), uE1-C, uE3, uFSH and uLH were significantly (*P* < 0.05) increased, but uPg and uCort were not different between females that ovulated compared to anovulatory females (Table 2). For uE1-C, uFSH and uLH, the difference between the two groups was almost exclusively due to the changes that were observed in hormone concentrations after DOD (Table 2). Within ovulating females, uE1-C, uE3 and uLH all had significantly (*P* < 0.05) increased mean concentrations post DOD (Table 2). Mean uFSH and uE3 were significantly higher in ovulating females compared to anovulatory females for all comparisons pre- and post-DOD, while no differences were detected within or between any group comparisons in mean uPg and uCort concentrations (Table 2).

### Urinary endocrine concentrations and follicle groups in ovulating female dolphins across the day prior to ovulation

**Figure 3:**
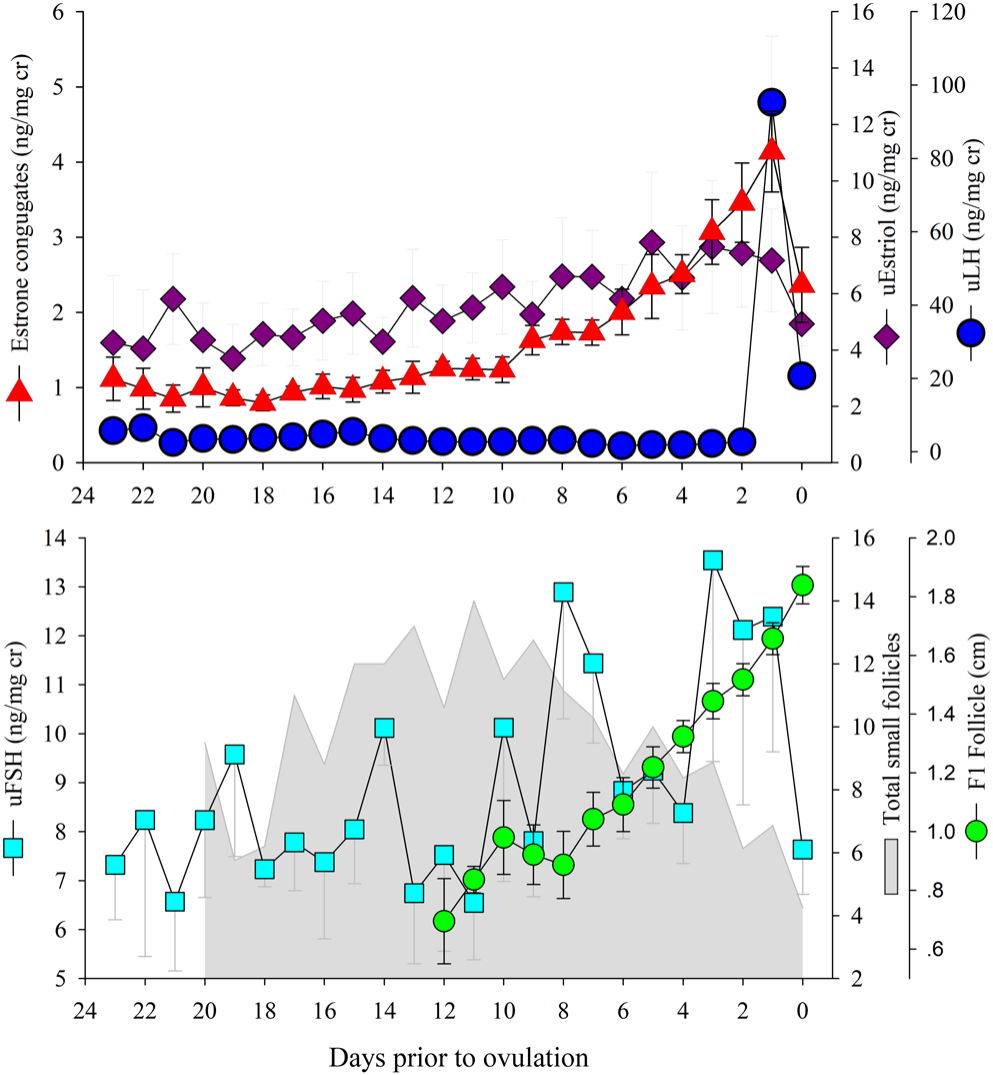
Top graph: daily measurements of urinary hormones. Estrone conjugate concentrations (red triangles) increased significantly (*P* < 0.001) daily until the day of ovulation. Estriol concentrations (purple diamonds) significantly increased until 3 days prior to ovulation. LH concentrations (blue circles) only increased prior to ovulation. Bottom graph: daily measurements of urinary FSH and ovarian follicles. No significant relationship was found between FSH and follicular size across all days prior to ovulation.

LMM regression demonstrated that uE1-C concentrations significantly increased (*P* < 0.001) across all days prior to ovulation, with significant relationships detected with tVSM (*P* = 0.027), tMD (*P* < 0.001) and tLG (*P* < 0.001) as follicles increased in numbers (Fig. 3). No significant effect was detected between uE1-C concentrations and TSM. Urinary E3 significantly increased (*P* < 0.001) across all days prior to ovulation; however, no significant association (*P* > 0.05) with any follicle group was detected. No significant relationship (*P* > 0.05) was detected between uFSH and DPO, tVSM, tSM, tMD or tLG follicles across all days prior to ovulation (Fig. 3). Urinary LH only significantly changed (increased) with increasing numbers of tLG follicles (*P* < 0.001). Significant, mildly marginal (mR^2^ ≥ 0.1) correlations were detected between tMD follicles and uE1-C, tLG follicles and uE1-C, tLG follicles and uLH, uE1-C and uE3 and uE1-C and uLH (Table 3). LMM analysis of these significant relationships pre- and post-DOD indicated that tMD (*P* = 0.01) and tLG (*P* = 0.001) only varied significantly with uE1-C after dominance (Table 4). In addition, uE3 was only correlated with uE1-C, and in a similar fashion, before and after dominance was established. As expected, uLH was significantly correlated with both tLG and uE1-C but only after DOD. All significant marginal correlations with uFSH were questionable (mR^2^ < 0.1), but they included both uE1-C and uPg before DOD and LH after DOD (Table 4).

**Table 3:** Follicle and urinary hormones pairwise correlations post-altrenogest treatment and across days prior to ovulation in bottlenose dolphins as represented by pairwise linear mixed model analysis with marginal and conditional correlation coefficients (mR^2^, cR^2^), respectively.

	tSM	tMD	tLG	E1-C	E3	Pg	FSH	LH
tVSM: 3–6 mm	NS	NS	NS	0.04, 0.51; (0.025)	NS	NS	NS	NS
tSM: >6–10 mm		NS	NS	NS	NS	−0.004,−0.51; (0.03)	NS	−0.02, −0.45; (0.04)
tMD: >10–16 mm			−0.04, 0.18; (0.017)	0.18, 0.27; (<0.0001	NS	NS	NS	NS
tLG: >16 mm				0.20, 0.57; (<0.0001)	NS	NS	NS	0.39*, 0.83 (<0.0001)
E1-C					0.31*, 0.50; (<0.0001)	NS	0.03, 0.33; (0.0005)	0.11, 0.34; (<0.0001)
E3						NS	0.02, 0.81; (0.004)	NS
Pg							0.05, 0.43; (0.0003)	0.05, 0.43; (0.001)
FSH								NS

Bold values indicate a mild (r ≥ 0.1) or greater marginal correlation. *P* values are provided within parentheses.

^*^Denotes a moderate marginal correlation (r ≥ 0.30).

E1-C, estrone conjugates; E3, estriol; FSH, follicle-stimulating hormone; LH, luteinizing hormone; NS, not significant (*P* > 0.05); Pg, progestagens; tLG, total large follicles; tMD, total medium follicles; tSM, total small follicles; tVSM, total very small follicles.

**Table 4:** Follicle and urinary hormones pairwise correlations post-altrenogest treatment before (B) a dominant follicle was identified (DOD) and after dominance was established (A) until ovulation in bottlenose dolphin as represented by pairwise linear mixed model analysis with marginal and conditional correlation coefficients (mR^2^, cR^2^), respectively.

DOD	tLG	E1-C	E3	Pg	FSH	LH
tVSM						
B	NA	**0.14, 0.41; (0.016)**	NS	NS	NS	−0.07, −0.57; (0.014)
A	−0.03, −0.51; (0.015)	0.06, 0.62; (0.002)	NS	NS	NS	NS
tSM						
B	NA	NS	NS	NS	NS	−0.09, −0.11; (0.045)
A	NS	NS	NS	−0.01, −0.49; (0.01)	NS	−0.03, −0.50; (0.015)
tMD						
B	NA	NS	NS	NS	NS	NS
A	−0.10, 0.15; (0.001)	0.08, 0.15; (0.01)	NS	NS	NS	NS
tLG^*^						
B	NA	NS	NS	NS	NS	NS
A		0.20, 0.57; (<0.0001)	NS	NS	NS	0.37, 0.84; (<0.0001)
E1-C						
B			0.30, 0.63; (<0.0001)	0.05, 0.56; (0.03)	0.06, 0.61; (0.0001)	NS
A			0.26, 0.46; (<0.0001)	NS	NS	0.10, 0.54; (<0.0001)
E3						
B				NS	NS	NS
A				NS	NS	NS
Pg						
B					0.02, 0.46; (0.027)	−0.02, −0.49; (0.028)
A					NS	NS
FSH						
B						NS
A						0.06, 0.47; (0.018)

Bold values indicate a mild (r ≥ 0.1) or greater marginal correlation.

^*^No large follicles were present before DOD, so the row was omitted.

aDOD, after dominance established; E1-C, estrone conjugates; E3, estriol; FSH, follicle-stimulating hormone; LH, luteinizing hormone; NA, not applicable because no large follicles were present; Pg, progestagens; PrDOD, prior to dominance; tLG: >16 mm, total large follicles; tMD: >10–16 mm, total medium follicles; tSM: >6–10 mm, total small follicles; tVSM: 3–6 mm, total very small follicles.

## Discussion

In the present study, 15 female bottlenose dolphins were examined, and 18 estrus synchronization treatment responses were analyzed using ovarian ultrasound imaging and urinary hormone assays. The results of these analyses have provided the first comparison of both hormonal and follicular changes between ovulating and non-ovulating post-altrenogest-treated females. We also have provided the first detailed description of follicular cohort growth patterns, including the identification of two follicular waves and the mean day for F1 deviation from the remaining follicular cohort from post-synchronization to ovulation. Finally, we described the relationships between multiple urinary reproductive hormones and changes observed in ovarian follicular activity.

A total of 58% of females placed on altrenogest responded to the treatment with post-withdrawal ovulation. This is in line with a previous report on bottlenose dolphins whereby only 48% (13 of 27) of the females responded to altrenogest treatment (Robeck *et al.* 2005). A 30% response (17 of 57) has also been observed in the Pacific white-sided dolphin (*Lagenorhyncus obliquidens*), a closely related delphinid (Robeck *et al.* 2009). Although it has been hypothesized that seasonal ovarian sensitivity plays a role in the lack of response to exogenous progestagen synchronization in bottlenose dolphins, no conclusive, supportive evidence has emerged (Robeck *et al.* 2018).

The urinary hormone concentration comparisons between these two groups, ovulators and non-ovulators, demonstrated that ovulating females had significantly greater mean concentrations of urinary FSH, LH and urinary estrogens (uE1-C, uE3), but no differences were detected in urinary Pg or cortisol. In an attempt to pinpoint the time when these hormone production differences occurred, possibly leading to a theory for this varied response, the relative changes in concentrations before and after the day of deviance (DOD) of the dominant follicle were evaluated. It was found that although FSH, the hormone commonly associated with follicular recruitment, was greater across both periods, it only significantly increased after DOD. Similarly, uE1-C, primarily representative of circulating E1 and E2, and uLH had significantly greater concentrations only after DOD. Both uFSH and uE3 had greater concentrations pre- and post-DOD in ovulating dolphins compared to those that did not ovulate.

The changes in uE1-C and uLH primarily after DOD were not unexpected because their production is stimulated via the well-documented hypothalamic–pituitary–ovarian (follicular) axis (Mariana 1991), a physiologic feedback loop that, as demonstrated within and elsewhere, clearly applies to bottlenose dolphins (Robeck *et al.* 2005, 2013). However, the role of E3 within this loop is unknown. Although uE3 significantly increased post-DOD compared to anovulatory females pre- and post-DOD, it differed from uE1-C in that its concentration only gradually increased toward ovulation and did not result in an obvious per-ovulatory surge. E3 is considered the least bioactive estrogen and is of primary importance during pregnancy (Kuiper *et al.* 1997). Bioactivity of E3 has been well described in primates, where it is believed to be produced via 16-hydroxylation of fetal dehydroepiandrosterone sulfate (Peter *et al.* 1994, Challis *et al.* 2000, Troisi *et al.* 2003). In bottlenose dolphins, it has been identified as the dominant (in concentration) estrogen during early pregnancy (Steinman *et al.* 2016), and its presence in significantly greater concentrations in ovulating females immediately post-altrenogest may indicate that it also plays a role in follicular recruitment. However, although not reported by the manufacturer, Steinman *et al.* (2016) discovered that the E3 antibody used in this study also has a high cross-reactivity with E2 (66.5%). As a result, it cannot be ruled out that the observations of E3 activity reported herein were solely due to E3 and not influenced by E2 concentrations. Regardless, no correlation between uE3 concentrations and follicle growth similar to a study where follicular fluid concentrations of E3 were evaluated in humans (Plaino *et al.* 2000). Interestingly, both uE3 (across the entire post-altrenogest DPO period) and uE1C (only before DOD) had significant positive correlations with uFSH, indicating a possible stimulatory role for these estrogens. Therefore, although E3’s role in the bottlenose dolphin estrous cycle appears to be independent of follicular growth, it may work in concert with E1 and E2 to provide the classic feedback stimulation of FSH production during early follicular recruitment.

FSH was significantly increased in ovulating females compared to non-ovulating dolphins prior to and after DOD. Current understanding of FSH’s role in follicular recruitment in other species supports the theory that the lack of gonadotropin-releasing hormone (GnRH) production was a primary reason for the lack of a secondary follicular wave that we observed (Richards 1979, Baird & McNeilly 1981, Bergfelt & Ginther 1985, Turzillo & Fortune 1990). Despite this observation, no significant relationship was found between follicle size and uFSH concentrations. However, a significant positive correlation between uFSH and uE1-C was detected but only prior to the deviation that coincided with the strongest (mR^2^ = 0.14) marginal correlation (as compared to any other follicle group except large) between VSM and uE1-C. The correlation between very small follicles and uE1-C, combined with a simultaneous uE1-C correlation with uFSH and the absence of a correlation between uE1-C and small follicles, provides us with indirect evidence that uFSH concentrations in dolphins may reflect biologic activity and, in conjunction with its indirect effect on estrogen production, its primary function in bottlenose dolphins is for follicular recruitment.

Although stress has been known to centrally inhibit or reduce GnRH release from the hypothalamus (Dobson & Smith 1995, Kalantaridou *et al.* 2010), urinary cortisol did not differ between the two groups, and therefore, stress most likely had a negligible influence on the different responses. Finally, genetic seasonality, which has been postulated to occur in bottlenose dolphins (Schroeder & Keller 1989), maybe the most likely explanation. This is based on non-latitudinally correlated seasonal changes in birth dates in wild bottlenose dolphins in the Gulf of Mexico that appear to coincide with seasonal food availability and not changes in the photoperiod (Urian *et al.* 1996). Because the animals in our study were first- or second-generation captive-born females, whose lineages would be from founders located in multiple oceanic regions, it is not known if this played a role in their response to treatment with altrenogest.

Urinary FSH appeared to be associated with two waves of small follicles with concentrations peaking around day 14 and day 10 prior to ovulation. These hormonal peaks were also followed by growth in the total number of small follicles, with both small follicle numbers and uFSH concentrations simultaneously decreasing as the dominant follicle reached 1.12 cm in size and 6 days prior to ovulation. These changes are similar to what has been observed in other mono-ovular species (equine and bovine), where, after follicular deviation, FSH is typically depressed from the follicular production of inhibin (Bergfelt *et al.* 1991, Adams *et al.* 1993, Rubianes & Menchaca 2003). In these species, and like the findings of this study (Fig. 2), small follicles stop developing and undergo regression over a few days after dominance has been established (Adams *et al.* 1992, Rubianes & Menchaca 2003).

In addition to these two possible waves of uFSH, we also observed a third increase which peaked 1 day prior to the LH surge and then dropped inversely as the LH surge occurred and 2 days prior to ovulation. This is similar to what has been previously reported for killer whale uFSH whereby a third peak of uFSH occurs just prior to LH peak (Robeck *et al.* 1993). Generally, in humans and domestic species, FSH is progressively depressed through combination of estrogen-modulated inhibin production, suppression of activin A, and differential receptor expression within follicles, as the dominant follicle approaches ovulation (Nett *et al.* 2002, Palermo 2007). In delphinids, due to the small number of medium and large follicles which are producing estrogens, this uncoupling of FSH and LH may not occur until just prior to ovulation when threshold levels of estrogen reach sufficient concentrations to exert its described effect within these species. In this study, we were unable to validate urinary inhibin or activin A assay, and thus, future work with serial serum samples may be the only way to define or detect when or if this feedback mechanism functions within cetaceans.

Constant presence (from day 22 to 1 day prior to ovulation) of very small follicles (3–6 mm, Fig. 2) was observed. Overall, in ovulating females, very small size follicles progressed to small follicles (6–10 mm) continually up until ovulation. Studies in other domestic animals have also observed the presence of small follicles throughout inter-ovulatory periods (Ginther & Pierson 1984, Pierson & Ginther 1984). For example, in mares, Ginther and Pierson (1984) observed follicles that were 2 mm in diameter for 10 days after ovulation, and in cattle, they were observed for 10 days prior to ovulation (Pierson & Ginther 1984). In non-ovulating bottlenose dolphins, after DOD, there were approximately half the number of very small follicles compared to ovulating females. And after ovulation, there were only one to two small follicles in the non-ovulating female group. Medium size (10–16 mm) follicles most often appeared approximately 5 days before ovulation, and the dominant follicle transitioned quickly (a few days) to a large size follicle (>16 mm), similar to what has been observed in cows (Pierson & Ginther 1984).

Within this study, it was observed that an initial post-altrenogest follicular wave did not culminate in the formation of a dominant follicle, similar to cattle that can have two to three follicular waves per cycle (Ginther *et al.* 1989, Adams *et al.* 1992, Burns *et al.* 2005). Follicles in the first wave regressed after reaching a medium size (between 6.1 and 10.0 mm). Conversely, during the second follicular wave initiated approximately 11 days prior to ovulation, the F1 follicle could be identified. In bottlenose dolphins, the period from altrenogest discontinuation to ovulation (~20 days) and the length of time where the F1 follicle can be identified prior to ovulation (~10 days) allows veterinarians and animal care managers the opportunity to observe the growth of the preovulatory follicle and to take action prior to the onset of the impending estrous cycle, i.e. physical separation of the female from a male in the same group to prevent copulation, preparation for timing of artificial insemination or moving the female to a group where breeding can occur.

In mares, a wave of follicles starts to develop before the previous ovulation (22 days) and grows continuously until reaching approximately 10 mm in size during the subsequent luteal phase before regressing (Pierson & Ginther 1987). This study only analyzed follicular growth prior to post-synchronized ovulation in female dolphins. Nonetheless, the mean number of very small follicles started to increase 2 days before ovulation for the animals that ovulated. In addition, a follicular wave of up to 10 mm in diameter peaked around day 6 post-altrenogest (16 days prior to ovulation) and declined at day 14 (8 days prior to ovulation). For bottlenose dolphins that ovulated, a follicular wave of very small follicles increased at the day of deviation (9.7 days prior to ovulation) and significantly decreased after that. An inverse correlation between predicted very small follicles and preovulatory follicle size was observed. This is supported by research in mares where after deviation, the dominant follicle increases in size, while the remaining follicles regress (Ginther *et al.* 2018).

In the present study, follicular dominance was established at 9.7 days prior to ovulation and from this point forward, the dominant follicle grew at a linear rate of 1.3 mm/day until ovulation at a mean size of 18.4 mm. This pattern of growth for the F1 follicle is similar to what has been described for other small delphinids including the Indo-pacific bottlenose dolphin (*Tursiops truncatus aduncus*) and the Pacific white-sided dolphin. For the Indo-pacific bottlenose dolphin, a 1.25 mm/day linear growth rate has been observed from day 12 until ovulation (Brook 1997). In Pacific white-sided dolphins, dominance is established when the follicle reaches 0.8 cm in diameter 6 days prior to ovulation with a linear growth rate of 0.12 cm/day (Robeck *et al.* 2009). For the largest delphinid, the killer whale, follicular growth rates of 0.98 cm/day with a mean POF size of 3.6 cm have been reported (Robeck *et al.* 2004). Interestingly, the growth rate of F1 observed in the small cetaceans mentioned above is essentially the same as what has been observed in cattle (1.2 mm/day), a species with an approximately 10-day shorter estrous cycle length (Ginther *et al.* 1989).

Within non-ovulating females, urinary FSH and LH were reduced compared to ovulating females across prior to and after DOD. This phenomenon of reduced gonadotropin concentrations that result in inadequate follicular recruitment and/or follicular growth toward deviance is also observed in mares during the transition phase from autumn to winter anestrus (Aurich 2011). Although the exact mechanism in bottlenose dolphins is not known, ovulation and calving occur in bi-seasonal peaks during the spring and fall with a large amount of individual variation, whereby some animals cycle throughout the year, while others have more pronounced periods of anestrus (Robeck *et al.* 2018). In horses, seasonality is controlled by the photoperiod (Freedman *et al.* 1979), but it has yet to be determined what, if any, environmental cue(s) regulate(s) individual seasonal ovarian activity in the bottlenose dolphin (Robeck *et al.* 2018). Besides differences in gonadotropin concentrations, non-ovulating females also had reduced concentrations of both uE1-C and uE3. It was assumed that these low concentrations could be explained by the reduced number of follicles of adequate size required to produce these steroid hormones.

Overall, this is the first detailed description of follicular and hormonal activity post-synchronization in bottlenose dolphins. The evidence presented herein supports the theory that dolphins experience two follicular waves, and it is during the second wave that the ovulatory follicle deviates from its cohort and increases in size until ovulation and a differential secretion of reproductive-associated hormones between ovulating and non-ovulating animals was also observed. Future research to determine if this pattern of follicular growth is also present during spontaneously cycling animals is warranted.

## Declaration of interest

The authors have no competing interests and SeaWorld Parks and Entertainment affiliations did not influence or alter our adherence to any Reproductive, Fertility and Development policies.

## Funding

This project was funded by SeaWorld Parks and Entertainment, Inc. Funder also provided support in the form of salary for authors G M, K S, T S, J O, M D and T R.

## Author contribution statement

G A M conceived the study, collected data, wrote the paper; P C, T L S and M R D collected data. K J S conceived the study, wrote paper, did laboratory assessment, analyzed data. J K O conceived and designed study, wrote paper. T R R conceived study, wrote paper, analyzed data, designed study. All authors approve the final submission of this manuscript.
